# CRISPR/Cas9-Mediated Generation of Guangxi Bama Minipigs Harboring Three Mutations in α-Synuclein Causing Parkinson’s Disease

**DOI:** 10.1038/s41598-018-30436-3

**Published:** 2018-08-20

**Authors:** Xiang-Xing Zhu, Yi-Zhi Zhong, Yao-Wen Ge, Ke-Huan Lu, Sheng-Sheng Lu

**Affiliations:** 1 State Key Laboratory for Conservation and Utilization of Subtropical Agro-Bioresources; Guangxi High Education Key Laboratory for Animal Reproduction and Biotechnology; College of Animal Science and Technology, Guangxi University, Nanning, 530004 China; 2 Guangxi Nanning Yanleshang Biotechnology Co. LTD, Nanning, 530004 China; 3 Wuhan ViaGen Animal Breeding Resources Development Company, Wuhan, 430073 China

## Abstract

Parkinson’s disease (PD) is a common, progressive neurodegenerative disorder characterized by classical motor dysfunction and is associated with α-synuclein-immunopositive pathology and the loss of dopaminergic neurons in the substantia nigra (SN). Several missense mutations in the α-synuclein gene *SCNA* have been identified as cause of inherited PD, providing a practical strategy to generate genetically modified animal models for PD research. Since minipigs share many physiological and anatomical similarities to humans, we proposed that genetically modified minipigs carrying PD-causing mutations can serve as an ideal model for PD research. In the present study, we attempted to model PD by generating Guangxi Bama minipigs with three PD-causing missense mutations (E46K, H50Q and G51D) in *SCNA* using CRISPR/Cas9-mediated gene editing combining with somatic cell nuclear transfer (SCNT) technique. We successfully generated a total of eight SCNT-derived Guangxi Bama minipigs with the desired heterozygous *SCNA* mutations integrated into genome, and we also confirmed by DNA sequencing that these minipigs expressed mutant α-synuclein at the transcription level. However, immunohistochemical analysis was not able to detect PD-specific pathological changes such as α-synuclein-immunopositive pathology and loss of SN dopaminergic neurons in the gene-edited minipigs at 3 months of age. In summary, we successfully generated Guangxi Bama minipigs harboring three PD-casusing mutations (E46K, H50Q and G51D) in *SCNA*. As they continue to develop, these gene editing minipigs need to be regularly teseted for the presence of PD-like pathological features in order to validate the use of this large-animal model in PD research.

## Introduction

Parkinson’s disease (PD) is the second most common progressive neurodegenerative disorder after Alzheimer’s disease, affecting more than 1% of the population aged over 60 years^1–3^. The cardinal neuropathological hallmark of PD is the selective loss of dopaminergic neurons from the substantia nigra (SN) pars compacta, as well as the presence of intracellular, proteinaceous inclusions called Lewy bodies (LBs) and Lewy neurites (LNs) in surviving neurons^2,4,5^. Progressive loss of the nigrostriatal projection results in a wide range of clinical motor symptoms, such as resting tremor, bradykinesia, muscle rigidity and postural instability.

Although most PD cases are sporadic, and aging is recognized as the greatest risk factor for the development of PD, about 10% of familial PD is currently ascribed to a monogenic mutation^1,3,6^. For example, *SCNA*, encoding the protein α-synuclein, was the first gene associated with inherited PD^7^. Whole-locus multiplications (duplications or triplications) and several missense mutations that occur in *SCNA* have been acknowledged as PD-causing mutations based on solid clinical evidence in humans^7–16^. It is worth mentioning that the identification of the first missense mutation in *SNCA*, A53T, led to the discovery of α-synuclein as the major component of LBs and LNs, the pathological hallmarks of PD^17^. This discovery was a turning point in the genetics and etiology of PD. The sequential development of α-synuclein-immunopositive pathology follows a fairly predictable order, which led Braak and colleagues to propose a universally recognized staging scheme for the progression of PD^18^. In addition, misfolding and aggregation of α-synuclein into neurotoxic species and cell-to-cell spread are currently the cornerstone of theories for the pathogenesis of PD^3–5^.

Animal models are valuable and necessary for PD research. In the past two decades, transgenic mouse models based on the expression of wild-type or mutant α-synuclein have been used to investigate the molecular basis and pathogenesis of PD^19–21^. However, few transgenic mouse models completely replicate the major pathologic features of human PD, such as true α-synuclein-immunopositive pathology and consistent dopaminergic neuron loss in the SN^19–21^. Additionally, PD is an age-dependent disorder caused by the slow development of LBs and LNs, which may take longer than the lifespan of a mouse to develop. Therefore, it is necessary to develop long-lifespan, large-animals models of PD.

Among the model organisms used in research, pigs, especially minipigs, are increasingly considered a superior large-animal model for human diseases^22–26^. Minipigs have a long lifespan (more than 10 years), similar body sizes to humans (compared to rodents), relatively short gestation periods (about 4 months), large litter sizes (more than 10 piglets per pregnancy), and puberty onset at 5 to 6 months. Furthermore, the neurophysiology and neuroanatomy of the minipig is similar to that of humans, making this species extremely useful for studies involving neurosurgery, brain imaging, and modeling of brain disorders by surgical and chemical methods^27–29^. For all these reasons, minipigs could serve as a suitable large-animal model for PD research.

In the present study, we generated Guangxi Bama minipigs harboring three PD-causing missense mutations (E46K, H50Q and G51D) in *SCNA*. To achieve this, we used CRISPR (clustered regularly interspaced short palindromic repeats)/CRISPR-associated endonuclease 9 (CRISPR/Cas9) gene editing system combined with the somatic cell nuclear transfer (SCNT) technique. The desired *SCNA* mutations were integrated into the genome, and successful transcription in gene-edited minipigs was confirmed by DNA sequencing. PD-specific pathological changes, including α-synuclein-immunopositive pathology and loss of SN dopaminergic neurons in mutant minipigs, were also examined by immunohistochemical analysis. We propose that these gene-edited minipigs carrying human PD-causing mutations can serve as an ideal large-animal model for PD research.

## Results

### Establishment of gene-edited cells

**Figure 1:**
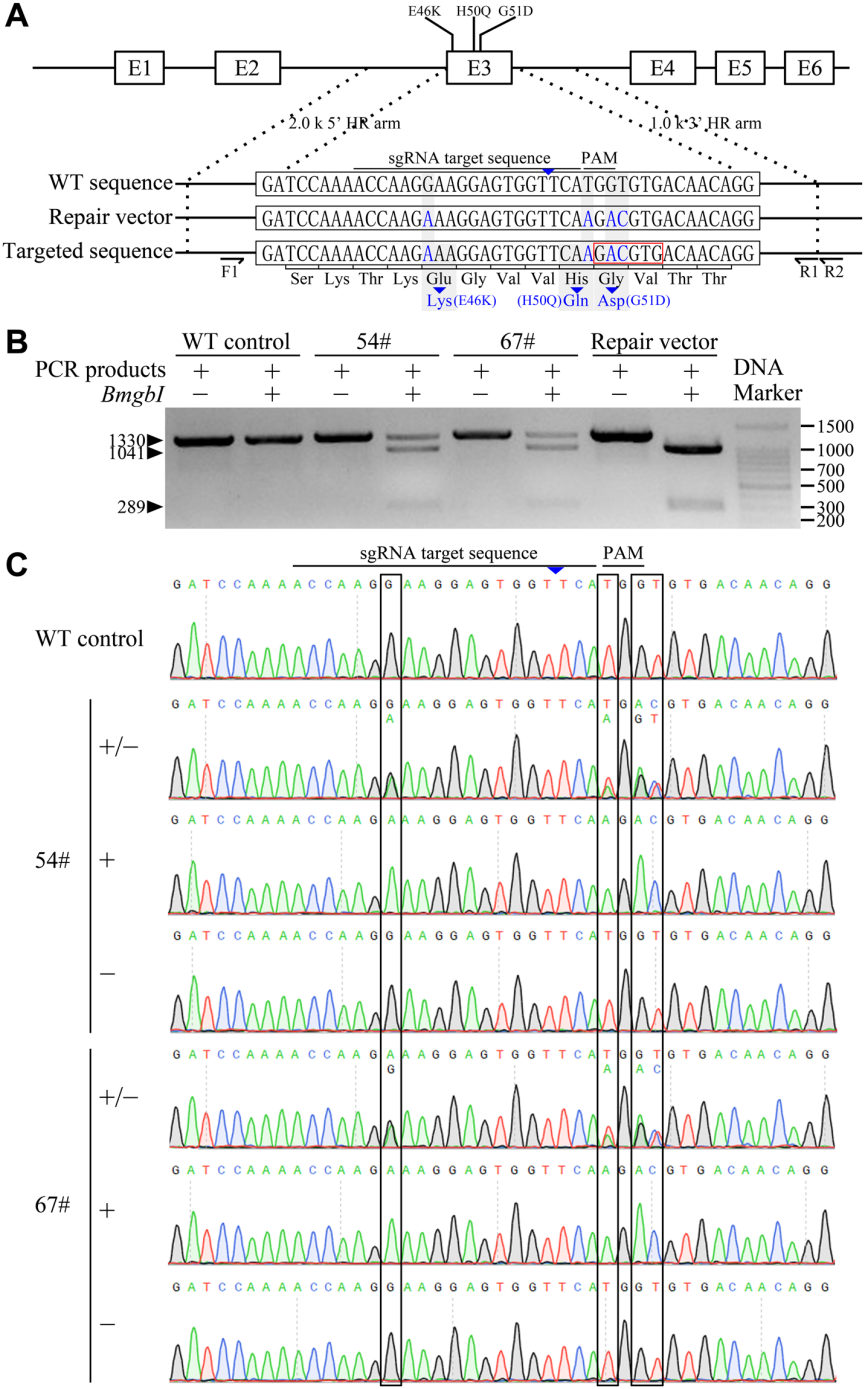
Establishment of α-synuclein gene-edited Guangxi Bama minipig fibroblasts. (**A**) Strategy for precise editing of *SCNA* by CRISPR/Cas9-mediated homologous recombination (HR) with repair vector. The schematic presents the cleavage site on exon 3 of the *SCNA* locus, the three Parkinson’s disease-causing missense mutations (E46K, H50Q, and G51D), the small guide RNA (sgRNA) sequence, and the protospacer adjacent motif (PAM) sequence. Blue letters indicate the designed mutations on the *SCNA* locus (targeted sequence) and the corresponding mismatched nucleotides in the repair vector. The wild-type (WT) sequence of the *SCNA* locus is also shown. The *Bmg*BI restriction enzyme site generated by the expected HR in the *SCNA* locus is denoted by a red box. (**B**) Cell colonies 54# and 67# carried monoallelic mutations in the *SCNA* locus and were identified by *Bmg*BI digestion. WT cells were used as a negative control and the repair vector as a positive control. (**C**) DNA sequencing further confirmed the presence of the three desired mutations in cell colonies 54# and 67#. WT cells served as the negative control.

To date, five different missense mutations in *SNCA*, namely A53T, A30P, E46K, H50Q and G51D, have been identified as causes of familial PD (see Supplementary Fig. S1). Upon aligning of the Bama minipig and human *SNCA* sequences (NCBI Gene No. 6622), we noticed that normal porcine α-synuclein contains a threonine at position 53 (see Supplementary Fig. S1). Therfore, we reasoned that the A53T substitution that causes PD in humans would not be similarly pathological in pigs. As shown in Fig. 1A, the E46, H50 and G51 amino-acid residues are located within exon 3 of *SCNA* in the Bama minipig, making it easy to simultaneously mutate all three residues with a single round of CRISPR/Cas9 gene editing. Accordingly, the E46K, H50Q and G51D PD-causing mutations were chosen as targets for modeling PD in Bama minipigs in this study.

The CRISPR/Cas9 plasmid and homologous recombination (HR) repair vector were designed (Fig. 1A) and co-transfected into fibroblasts through Xfect transfection. After resistance selection with puromycin for 5 days, we found that only a few cells survived. However, when puromycin was withdrawn, followed by cell culture for 7 to 10 days, many individual cell colonies were generated. A total of 166 cell colonies were picked up for proliferation, of which 94 cell colonies were expanded. After then, genotyping by *Bmg*BI digestion and DNA sequencing were performed to confirm that the desired gene editing had successfully occurred in the cells. As shown in Fig. 1B, cell colonies 54# and 67# (2.1%) showed positive digestion by *Bmg*BI. Furthermore, DNA sequencing also confirmed that the cell colonies 54# and 67# contained three monoallelic PD-causing missense mutations (E46K, H50Q and G51D) in *SCNA* (Fig. 1C).

The gene-edited cell colonies 54# and 67# proliferated well. Since the integration of exogenous genes could confuse the phenotype of genetically modified animals, we performed PCR amplification to examine whether any exogenous genes had been integrated into these two gene-edited cell colonies. The PCR results showed that no exogenous genes, specifically the CRISPR/Cas9 plasmid and the repair vector, were integrated into cell colonies 54# and 67# (see Supplementary Fig. S2).

Off-target effect is another concern when CRISPR/Cas9 is used for cellular genome editing. Three off-target sites (OTSs) were detected in the gene-edited cell colonies with potential off-target effects (see Supplementary Table S1). DNA sequencing showed that no mutation had occurred in any of the detected OTSs in either cell colony (see Supplementary Fig. S3). Having confirmed precise, site-specific modifications without exogenous gene integration or off-target mutations, the cell colonies 54# and 67# were ready to be used in the production of gene-edited minipigs.

### Production of cloned minipigs

**Figure 2:**
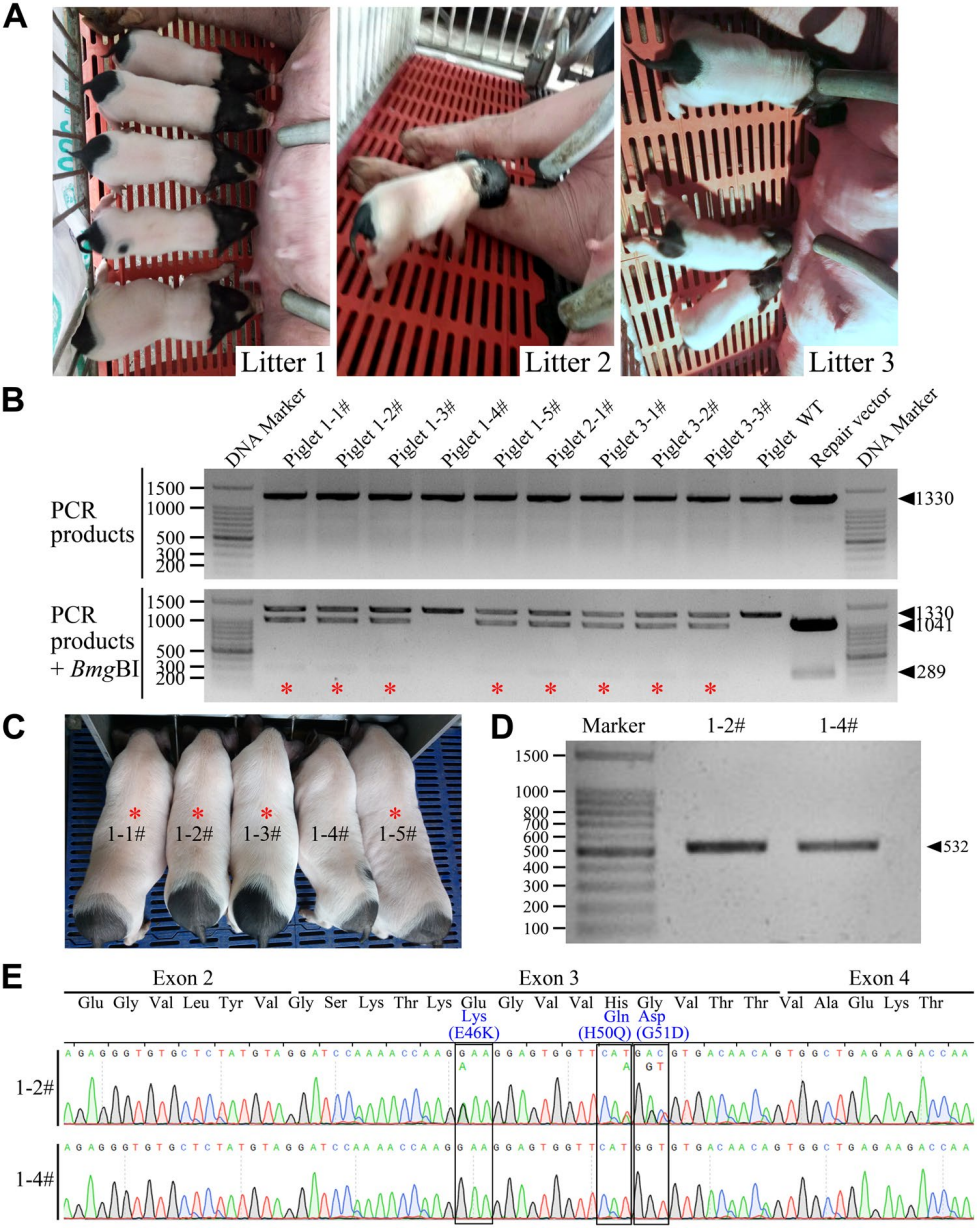
Production and genotyping of gene-edited Guangxi Bama minipigs. (**A**) A total of nine cloned piglets from three litters appeared healthy at birth. (**B**) *Bmg*BI digestion analysis showed that eight of nine piglets were monoallelic mutant at the *SCNA* locus (labeled by red asterisks), and the remaining one (1–4#) was a wild-type (WT). WT minipig were used as a negative control and the repair vector as a positive control. (**C**) Genotype-confirmed mutant minipigs (labeled by red asterisks) and the one wild-type minipig remained clinically healthy and showed normal growth and development at 3 months of age. (**D**) One mutant minipig (1–2#) and the age-matched wild type (1–4#) were used to confirm the *SCNA* mutations at the transcriptional level. The *SCNA* mRNA-coding sequence was obtained by RT-PCR. (**E**) The presence of the desired mutations at the transcriptional level (sequence coding for *SCNA* mRNA) was confirmed by DNA sequencing.

After genotyping, exogenous gene detection, and off-target analysis, the gene-edited cell colonies 54# and 67# were used as donor cells to produce cloned minipigs. A total of 1,563 SCNT embryos were transferred into six surrogate recipients. Early pregnancy was monitored in four surrogates, three went to full term and the last aborted on day 31 after embryo transfer. Eleven live male piglets and two stillborn piglets were delivered from the three surrogates via natural delivery (Table 1). The overall cloning efficiency was 0.83% (13/1,563). Two piglets with abnormal development died soon after birth. The remaining nine cloned piglets appeared healthy at birth (Fig. 2A) and developed normally after then, indicating that the gene editing of donor cells did not significantly impair the survival or health of the SCNT-derived Bama minipigs.

**Table 1:** Production of gene-edited Guangxi Bama minipigs via somatic cell nuclear transfer.

Cell colony	Recipient No.	No. of embryos transferred	Day 40 pregnancy status*	Gestation period (day)	No. of piglets delivered (alive/stillbirth)	Cloning efficiency (%)**
54#	LY16615	206	+	118	5/1	
54#	LY166091	250	Returned to estrus			
54#	LY114501	194	+	116	2/1^#^	
67#	LY16711	309	Returned to estrus			
67#	Y37	301	+	117	4^##^	
67#	LY16144	303	Aborted at Day 31			
	Total	1,563			11/2	0.83

^*^Pregnancy status: + , pregnant.

^**^Cloning efficiency: No. of piglets born/No. of embryos transferred × 100%.

^#,##^One live-born piglet had severe developmental abnormalities that caused its death soon after birth.

### Genotyping and *SCNA* mRNA-coding sequence analysis

Genomic DNA extracted from tail biopsies of all live cloned piglets was collected. PCR amplification was performed, following digested with *Bmg*BI for genotyping. As shown in Fig. 2B, eight of the nine live cloned piglets (88.9%) were monoallelic mutants (*Bmg*BI digestion-positive), and the remaining one (1–4#) was a wild-type. DNA sequencing further confirmed that the desired PD-causing mutations were successfully introduced into one allele of the *SCNA* gene in the eight SCNT-derived piglets (see Supplementary Fig. S4).

Having confirmed the genotype of the nine cloned piglets, we next verified the presence of the desired mutations in the *SCNA* mRNA-coding sequence. Since *SCNA* is specifically expressed in the central nervous system of pigs, one of the genotype-confirmed mutant minipig (1–2#) and a wild-type control (1–4#) from the same litter were euthanized at 3 months of age for mRNA analysis (Fig. 2C). The brain tissue of the mutant and wild-type minipig was collected, the total RNA was extracted, and then RT-PCR was performed. The resultant cDNA was PCR amplified to produce the *SCNA* mRNA-coding sequence (Fig. 2D), which was then verified by DNA sequencing. As shown in Fig. 2E, DNA sequencing analysis confirmed that the three mutations were present in the *SCNA* mRNA-coding sequence of the gene-edited minipigs. These results indicated that we had successfully generated Guangxi Bama minipigs harboring three heterozygous PD-causing mutations in *SCNA* using CRISPR/Cas9-mediated gene editing combined with the SCNT technique.

### Analysis of PD-specific pathological changes

**Figure 3:**
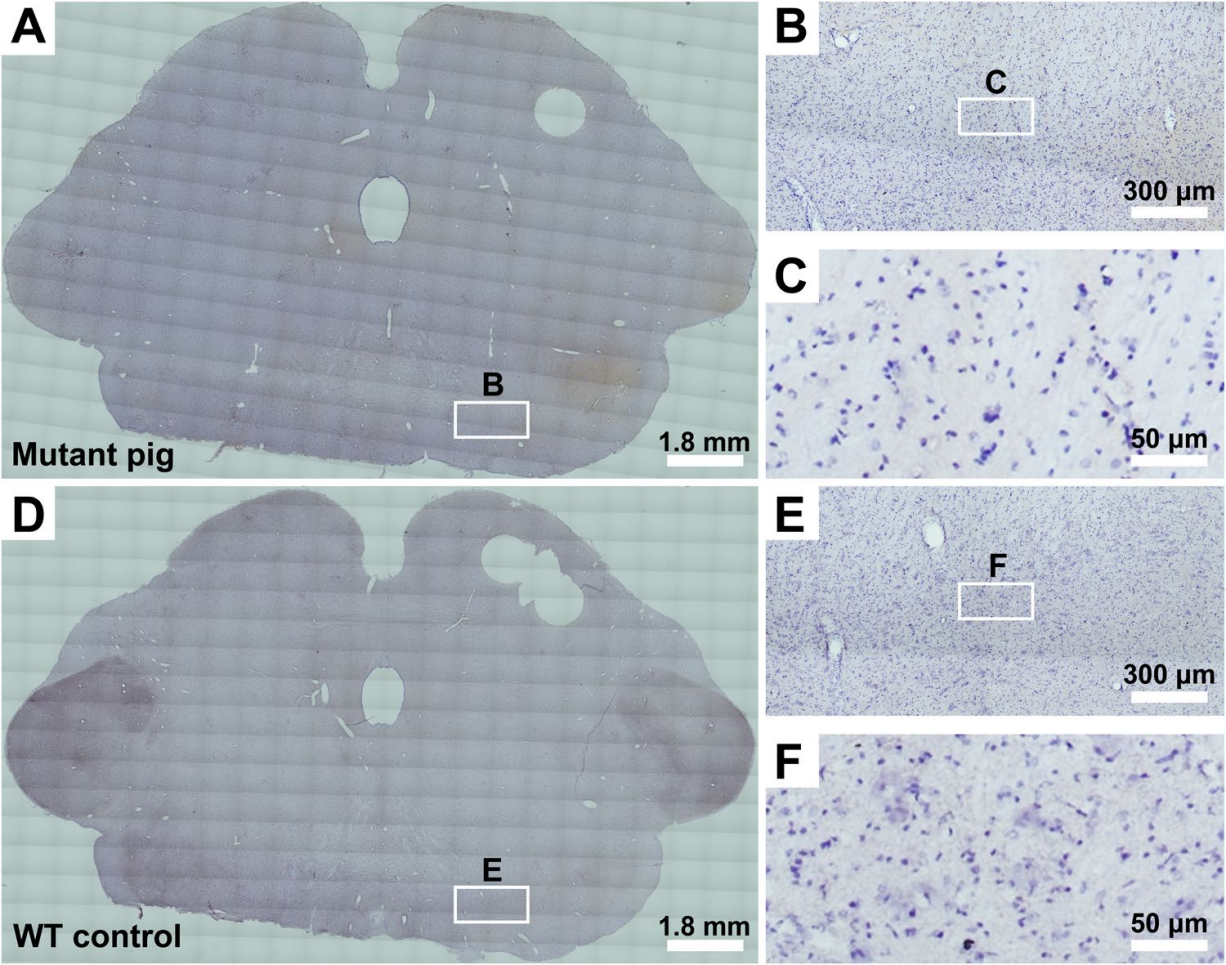
Detection of Parkinson’s disease (PD)-specific pathological changes in a mutant Guangxi Bama minipig. (**A–C**) One 3-month-old mutant Guangxi Bama minipig (1–2#) was used to detect PD-specific pathological changes in the substantia nigra by α-synuclein immunohistochemical staining. (**D–F**) One age-matched wild-type (WT) minipig (1–4#) was used as a negative control. (**B,C,E,F**) Higher-magnification images show no classical α-synuclein-immunopositive pathology in the substantia nigra of the mutant minipig at 3 months of age (**B,C**) compared to the age-matched WT minipig (**E,F**). Note that the immunohistochemistry was performed on the brain tissue from the same two minipigs that were euthanized for mRNA analysis.

**Figure 4:**
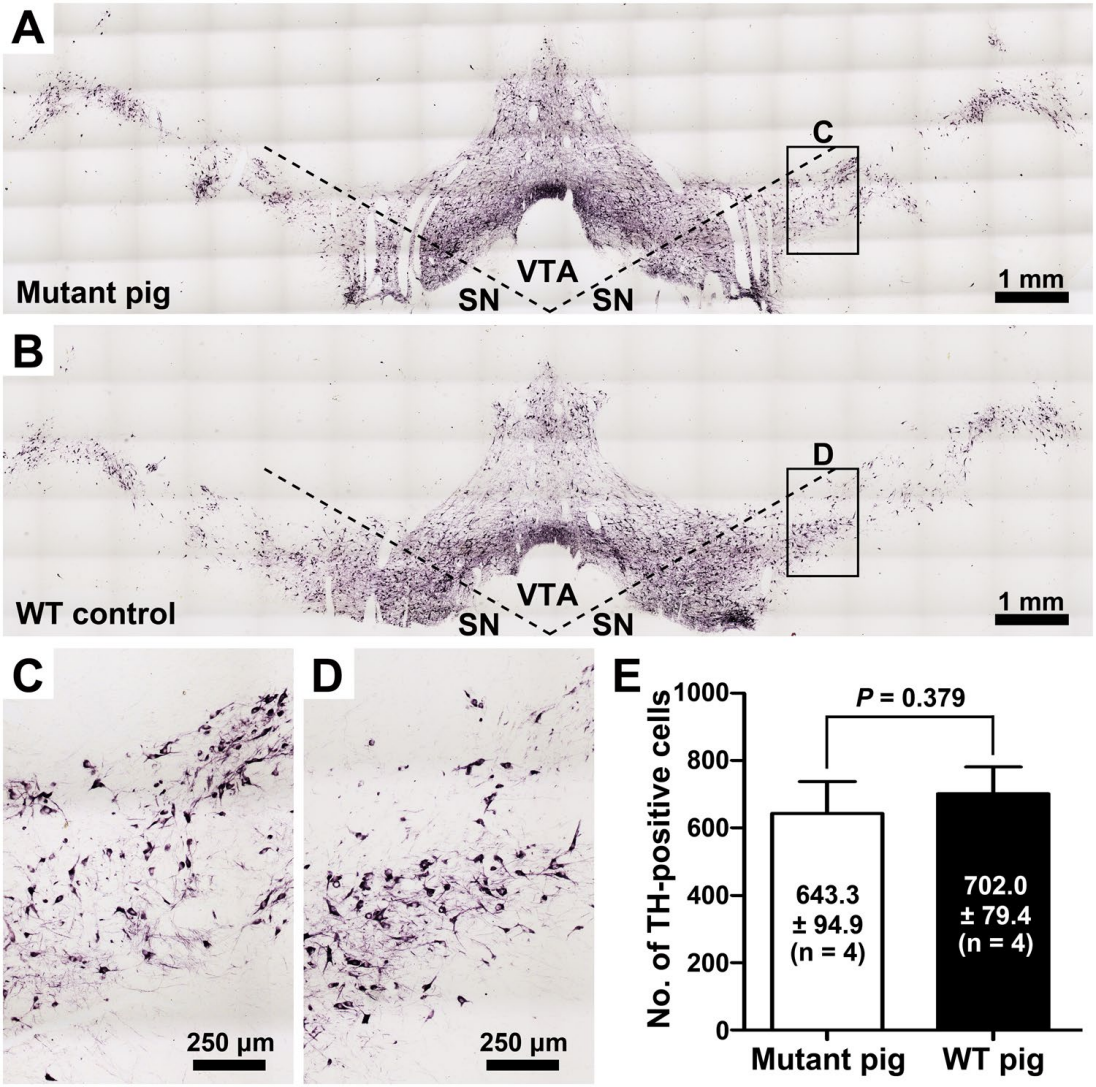
Detection of dopaminergic neuron loss in the substantia nigra of a mutant Guangxi Bama minipig. (**A–D**) A mutant Guangxi Bama minipig (1–2#; **A** and **C**) and a wild-type (WT) control (#1–4; **B** and **D**) at 3 months of age were used for tyrosine hydroxylase (TH) immunohistochemical staining of the dopaminergic neurons in the substantia nigra. At least three randomly selected sections from approximately the same anatomic parts of mesencephalon were examined. (**E**) The number of dopaminergic neurons (No. of TH-positive cells) are expressed as the mean ± standard deviation (SD), and differences between the two minipigs were analyzed by two-tailed Student’s *t*-test. The statistical analysis showed that there was no significant difference between the mutant minipig and WT control at 3 months of age. Note that the immunohistochemistry was performed on the brain tissue from the same two minipigs that were euthanized for mRNA analysis. (SN, substantia nigra; VTA, ventral tegmental area).

Eight gene-edited Guangxi Bama minipigs were clinically healthy and showed normal growth and development. However, when they reached 3 months of age, none of these minipigs exhibited PD-like symptoms, such as resting shaking, muscle rigidity, slowness of movement, or difficulty walking. To determine whether the PD-specific pathological changes, such as α-synuclein-immunopositive pathology and loss of dopaminergic neurons in the SN, had occurred, the mutant minipig (1–2#) was euthanized (at 3 months of age) for immunohistochemical analysis. The wild-type minipig (1–4#) from the same litter was served as a negative control. Note that the immunohistochemistry was performed on the tissue from the same two minipigs that were euthanized for mRNA analysis described above. As shown in Fig. 3, no classical α-synuclein-immunopositive pathology was detected from the SN of the mutant minipig. In addition, quantitative analysis of dopaminergic neurons in the SN indicated that there was no significant loss of dopaminergic neurons in the mutant minipig compared to the age-matched wild-type minipig (643.3 ± 94.9 cells in the mutant vs. 702.0 ± 79.4 cells in the wild-type, *P* = 0.379; Fig. 4). These results indicated that neither of the PD-specific clinical and pathological changes had occurred in gene-edited minipigs at 3 months of age, despite the fact that they harbored three *SCNA* mutations (E46K, H50Q, and G51D) that cause PD in humans.

## Discussion

Genetically modified pigs have been widely used to model different human diseases because this species possesses many physiological and anatomical similarities to humans^22–26^. However, precise genetic modification of the pig genome is challenging. The first approach used to modify the pig genome was pronuclear injection of DNA into pig zygotes. Pronuclear injection-mediated transgenesis is very inefficient, with only about 1% of injected zygotes producing transgenic piglets^23,25^. In addition to genetic engineering by pronuclear injection, other techniques, such as sperm-mediated transfection and intracytoplasmic sperm injection (ICSI)-mediated transgenesis have been developed to produce genetically modified pigs. However, each of these techniques results in random gene integration and is unable to achieve precise gene modification through HR^23,25^. In contrast, genetic modification of somatic cells followed by SCNT offers the considerable advantage of confirming the desired genetic modification prior to generating the pigs^22–25^. Furthermore, since no germline-competent embryonic stem cells have been generated for gene targeting in pigs, HR in somatic cells followed by SCNT has become the primary strategy for creating gene knock-out (KO) and gene knock-in (KI) pigs^22–25^. Still, the efficiency of traditional HR in somatic cells is extremely low, thereby greatly hindering the generation of precise genetically modified pigs^22–25^.

Recently, the emergence of gene-editing nucleases, especially the revolutionary CRISPR/Cas9 system, has greatly enhanced the efficiency of precise gene editing in various mammalian species. The CRISPR/Cas9 system has been used to efficiently generate gene-edited pigs by either direct injection of CRISPR/Cas9 mRNA into zygotes produced *in vivo* or genetic modification of somatic cells followed by SCNT^24,25^. This valuable technical advance significantly facilitated the creation of genetically modified pig animal models of human genetic diseases.

In the present study, we aimed to generate Guangxi Bama minipigs harboring PD-causing mutations in *SCNA* by using CRISPR/Cas9-mediated gene editing in somatic cells followed by SCNT of the gene-edited cells. The Guangxi Bama minipig is a unique miniature pig strain that originates from the Bama County of Guangxi province, China. This minipig is characterized by its small body size (adult body mass of approximately 50 to 60 kg), high degree of inbreeding, ease of handling, and physiological and anatomical similarities to humans^30–36^. Many genetically modified Guangxi Bama minipigs have already been generated and have served as ideal tools to study human diseases such as von Willebrand disease^37^, Waardenburg syndrome^38^, ectodermal dysplasia-9^39^, and severe combined immune deficiency^40^. In addition, gene-edited Guangxi Bama minipigs have been used to model PD by targeting other PD-associated genes, such as *Parkin*, *DJ-1* and *PINK1*^41–43^. For all these reasons, we propose that Guangxi Bama minipigs can serve as an ideal model for PD research.

Successful gene editing of somatic cells is the first step to generating genetically modified pigs via SCNT. In a preliminary experiment, we first examined the efficiency of double-stranded DNA cleavage induced by the CRISPR/Cas9 system. Using the carefully designed sgRNA, we obtained 20% of cell colonies (2/10) carried indel mutations generated by non-homologous end joining (NHEJ)-mediated DNA repair, confirming the efficiency of the CRISPR/Cas9 system for gene targeting in Guangxi Bama minipig somatic cells (data not shown). However, after co-transfectiond of somatic cells with the CRISPR/Cas9 plasmid and HR repair vector, only 2.1% (2/94) of the detected cell colonies carried monoallelic KIs of the desired PD-causing *SCNA* mutations generated by HR-mediated DNA repair. The KI efficiency obtained in our study was lower than that achieved by Han *et al*. Han *et al*.^39^, Lai *et al*.^44^ and Yang *et al*.^45^, who reported 18.7% (32/137), 11.4% (21/184) and 5.6% (5/90) gene editing efficiencies, respectively. The lower gene editing efficiency in our study could be caused by differences in the cell line, target sequence, HR repair template and genetic modification method.

Differences in resistance selection strategy could also play an important role in gene editing efficiency. Normally, colony selection via antibiotic resistance is used to screen for targeted cells that have been successfully transfected with CRISPR/Cas9 plasmid and HR repair vector. Persisting resistance selection (continued incubation with the antibiotic drug) until the formation of cell colonies may result in a higher efficiency of gene editing, but may also lead to exogenous gene integration and off-target effects. The integration of exogenous genes and off-target mutations can cause unexpected biological effects and should thus be avoided when generating genetically modified pigs^37–45^. Accordingly, we maintained resistance selection for only five days after transfection. Retraction of resistance selection permits the growth of transfected cells without effective anti-drug gene expression, resulting in the formation of unedited cell colonies. Using our resistance selection strategy, we obtained two gene-edited cell colonies and confirmed that no exogenous gene integration and no off-target mutations existed in the detected OTSs. Furthermore, by using these precisely modified cell colonies as donor cells, we successfully generated nine live cloned Bama minipigs. Genotyping analysis revealed that eight (88.9%) of the cloned minipigs were monoallelic mutants and only one was a wild type. The generation of this wild-type pig could be due to the presence of a few wild-type cells in the selected colonies, an occurrence also reported by other researchers^39,42^. Therefore, ensuring precise gene editing in donor cells and then using these cells to generate genetically modified pigs through SCNT technology ensures that all the offspring carry the desired genetic modifications and reduces the cost of producing large animals.

So far, five different missense mutations in *SCNA*, namely A53T, A30P, E46K, H50Q and G51D, have been identified as causes of familial PD^1,6^. PD patients with these missense mutations present classical PD symptoms and α-synuclein-immunopositive pathology^7–16^. We noticed that porcine α-synuclein normally contains a threonine at position 53, indicating that the A53T mutation is only pathogenic within the human context^1,5,6^. Although there are many transgenic mouse models based upon expression of mutant α-synuclein, few of them can fullly replicate the major pathological features of human PD^19–21^. Clinically, PD is an age-dependent disorder caused by the development of α-synuclein-immunopositive pathologies, which might take longer than the lifespan of a mouse to develop. Therefore, we posited that Guangxi Bama minipigs harboring PD-causing, human *SCNA* mutations can be a suitable large-animal model for PD research.

In our study, the gene-edited minipigs remained clinically healthy, and we did not detect PD-like symptoms such as shaking at rest, muscle rigidity, slow movement, or difficulty walking in 3-month-old mutant Bama minipigs. Moreover, we did not observe any classical α-synuclein-immunopositive pathology or loss of dopaminergic neurons in the SN by immunohistochemical analysis of the 3-month-old mutant minipig. Two previous studies reported that gene-edited Guangxi Bama minipigs created by targeting other PD-associated genes such as *Parkin*, *DJ-1* and *PINK1*, did not show PD-like symptoms even when they reached seven to ten months of age^42,43^. Even in carriers of *Parkin*, *DJ-1* and *PINK1* mutations, the PD symptoms often appeared at the age of 20 to 40 years^7–16^. In line with this finding, the results of our study demonstrated that a few months is not sufficient time for a minipig to develop PD-like pathological changes in the brain.

In summary, in the present study, we successfully generated gene-edited Guangxi Bama minipigs harboring three heterozygous, PD-causing *SCNA* mutations by using CRISPR/Cas9-mediated gene editing combined with the SCNT technique. However, we did not observe α-synuclein-immunopositive pathology or loss of SN dopaminergic neurons (PD-specific pathological brain changes) in the gene-edited minipigs at 3 months of the age. These genetically modified animals need to be continually tested for the appearance of PD-like pathological features that would validate the Guangxi Bama minipig as a large-animal model of PD.

## Methods

### Animal ethics

All animal procedures used in this study were carried out in accordance with the *Guide for Care and Use of Laboratory Animals* (8th edition, released by the National Research Council, USA) and were approved by the Animal Care & Welfare Committee of Guangxi University (GXU2016-013). Pig ovaries used in the *in vitro* production of mature oocytes (SCNT recipients) were collected from a slaughterhouse. All animal surgical procedures were performed under anesthesia by a veterinarian, and all efforts were made to minimize animal suffering.

### Reagents and chemicals

Unless otherwise stated, all organic and inorganic reagents were purchased from Sigma-Aldrich Co. (St. Louis, MO, USA). All solutions that were made in-house were filtered through a 0.22 μm filter (Millipore, Bedford, MA, USA) and stored at 4 °C or −20 °C until use. All pipette tips, centrifuge tubes, and petri dishes were single-use, disposable equipment purchased in sterile packaging.

### Construction of the CRISPR/Cas9 plasmid and the homologous recombination (HR) repair vector

Design and construction of the CRISPR/Cas9 plasmid were performed according to the method described by Ran *et al*.^46^. The sgRNA used to target exon 3 of *SCNA* (NCBI gene ID: 641350) was ACCAAGGAAGGAGTGGTTCATGG (the protospacer adjacent motif [PAM] sequence is underlined), designed using the CRISPR Design Tool (http://tools.genome-engineering.org; Fig. 1A). The pSpCas9(BB)−2A-Puro (PX459) V2.0 plasmids (Addgene #62988) were linearized by *Bsb*I restriction enzyme digestion and linked with the annealed sgRNAs using a T4 DNA Ligase (Takara, Dalian, China). The fully constructed CRISPR/Cas9 plasmid was confirmed by DNA sequencing (BGI, Shenzhen, China).

For construction of homologous arms, a 3k bp sequence arounding the targeting locus was amplified by genomic PCR using the primers PF: 5′-GACTCAGACCAGTGTTTCTC-3′ and PR1: 5′-CTAAGGTCACCACAGCTGTC-3′. Mismatched nucleotides leading to the three PD-causing missense mutations (E46K, H50Q, and G51D) were introduced by using the PCR-driven overlap extension method described by Heckman and Pease^47^. The design incorporated a *Bmg*BI restriction enzyme site (Fig. 1A) that was used for confirmation of gene editing by restriction enzyme digestion. Finally, the 3k bp homologous sequence harboring the desired mutations was cloned into the pMD18-T Vector (Takara) and confirmed by DNA sequencing.

### Transfection and selection of gene-edited cell colonies

The procedures used for isolation, cultivation, and transfection of newborn Guangxi Bama minipig kidney fibroblasts were based on our previous studies^33,48,49^. Once deeply anesthetized, the piglet was euthanized, and both kidneys were dissected out and minced in Dulbecco’s phosphate-buffered saline (DPBS; Gibco, Grand Island, NY, USA). Tissue fragments were washed several times with DPBS and digested in 0.25% (w/v) trypsin-EDTA solution for 30 min at 37 °C. Isolated cells were cultured for 1–2 passages in Dulbecco’s modified Eagle’s medium (DMEM; Gibco) supplemented with 15% (v/v) fetal cattle serum (FCS; Gibco) and then frozen in liquid nitrogen until use.

Two days before transfection, the frozen cells were thawed and cultured without antibiotics in a 35-mm cell culture dish (NUNC) until they were at approximately 50% confluence. The cell culture medium was replaced with fresh medium, into which we added the polyplexes prepared with 1.5 µL Xfect polymer (Clontech, Palo Alto, USA), 2.5 µg CRISPR/Cas9 plasmid, and 2.5 µg HR repair vector. These reagents were incubated overnight at 37 °C in a humidified atmosphere of 5% (v/v) CO_2_ in air. At 24 h post-transfection, cells were split into eight six-well cell culture clusters (NUNC). After 24 h of recovery, the transfected cells underwent resistance selection with 0.8 µg/mL puromycin (Solarbio, Beijing, China) for 5 days, after which puromycin was withdrawn, and the cells continuously cultured for 7–10 days. Individual cell colonies were picked up and cultured in 24-well cell culture clusters (NUNC). When confluence was achieved, the cell colonies were subcultured and some collected for genotyping. Gene-edited cell colonies were expanded and then cryopreserved.

### Genotyping

Genomic DNA was extracted from cell colonies and tail tissue of newborn cloned piglets using a TIANamp Genomic DNA Kit (Tiangen, Beijing, China). PCR reactions were conducted with 2 µL of genomic DNA, 0.5 µL forward primer (10 μM), 0.5 µL reverse primer (10 μM), 10 µL PrimeSTAR Max (Takara), and deionized water to make a total volume of 20 µL.

A specific primer pair was designed to amplify the homologous short arm (PF1: 5′-AAGTGAGACAGGAGAGGTTG-3′; PR2: 5′-TGTGACTGCGGAACCAAAAC-3′), resulting in a 1,330 bp amplicon (Fig. 1A). PCR amplification conditions were as follows: one cycle at 95 °C for 5 min; 35 cycles at 98 °C for 10 sec, 56 °C for 15 sec, and 72 °C for 10 sec, followed by 72 °C for 5 min. The PCR products were run on a 1% (w/v) agarose electrophoresis gel containing 0.01% (v/v) Andy Gold Nucleic Acid Gel Stain (Applied BioProbes, Davis, CA, USA), purified from the gel, and digested with the *Bmg*BI restriction enzyme (Takara). If the three desired mutations were correctly generated by gene editing, the 1,330 bp PCR product was cut by *Bmg*BI into two fragments of 1,041 bp and 289 bp (Figs 1B; 2B). As a positive control for *Bmg*BI digestion, the HR repair vector was amplified by PCR (using the HR repair vector as the PCR template) using the primers designed to amplify the HR short arm (PF1: 5′-AAGTGAGACAGGAGAGGTTG-3′; PR1: 5′-CTAAGGTCACCACAGCTGTC-3′), resulting in a 1,301 bp amplicon (Fig. 1A). Upon *Bmg*BI restriction enzyme digestion, this positive-control PCR product generated two fragments of 1,012 bp and 289 bp (Figs 1B and 2B). Samples with the correct *Bmg*BI digestion fragments were collected for DNA sequencing to confirm whether the desired genetic modifications had occurred at the *SCNA* locus.

### Detection of exogenous gene integration in gene-edited cell colonies

Integration of the CRISPR/Cas9 plasmid and HR repair vector in gene-edited cell colonies was detected by PCR using specific primers. For the CRISPR/Cas9 plasmid, the primers were PF: 5′-ATGGACTATAAGGACCACGA-3′ and PR: 5′-TATCCTCTTCCACCAGGAAG-3′. For the HR repair vector, the primers were PF: 5′-CCGAGCTCGAATTCGTAATC-3′ and PR: 5′-GCCTGGTATCTTTATAGTCC-3′. Glyceraldehyde-3-phosphate dehydrogenase (*GAPDH*) served as the reference gene with the PCR primers PF: 5′-TCTGCATCAGTGCTCCTTGA-3′ and PR: 5′-AAGAGGTGATGAAGCTCCGA-3′. Cell colonies that integrated either the CRISPR/Cas9 plasmid or HR repair vector were not used for SCNT.

### Off-target site (OTS) detection in gene-edited cell colonies

Potential OTSs were predicted by the CRISPR Design Tool (http://tools.genome-engineering.org) according to previously described methods^37,39,43,46^. Three sites with potential off-target effects (see Supplementary Table S1) were selected for testing in the gene-edited cell colonies. Specific primers (see Supplementary Table S1) were used for PCR, and the products were sequenced to confirm whether off-targeting mutations existed. Off-target mutations were identified by alignment of sequenced alleles to the known wild-type allele. Cell colonies harboring any off-target mutations were not used for SCNT.

### Production of mutant minipigs via SCNT

The procedures used for donor cell preparation, *in vitro* maturation of porcine oocytes, SCNT, and embryo transfer were conducted according to our previous studies^34,48,49^.

Gene-edited cell colonies that had no exogenous gene integration or off-target mutations were used to generate mutant minipigs. In order to prepare SCNT donor cells, cryopreserved mutant cells were thawed and cultured for two days, followed by synchronization via serum starvation (DMEM supplemented with 0.5% FCS) for 48 h. The cells were then harvested and re-suspended in 1 mL micromanipulation medium (10 mM HEPES-buffered TCM-199 containing 0.3% [w/v] bovine serum albumin [BSA]; pH = 7.3). This cell suspension was maintained at room temperature and used as the source of SCNT donor cells. Cumulus-oocyte complexes (COCs) were aspirated from the follicles and washed twice in PVA-TL-HEPES medium. The COCs were transferred into 200 µL drops of preheated maturation medium (bicarbonate-buffered TCM-199 supplemented with 0.1% [w/v] polyvinyl acetate [PVA], 3.05 mM D-glucose, 0.91 mM sodium pyruvate, 0.57 mM cysteine, 10 ng/mL epidermal growth factor [EGF], 0.5 µg/mL follicle-stimulating hormone [FSH], 0.5 µg/mL luteinizing hormone [LH], 0.0750 g/L penicillin G, 0.0500 g/L streptomycin and 10% [v/v] porcine follicular fluid), covered with mineral oil, and then incubated for 20–22 h at 38.5 °C in a humidified atmosphere of 5% (v/v) CO_2_ in air. Next, the COCs were cultured for an additional 20 h in the same maturation medium without the gonadotropins. Following maturation, expanded cumulus cells were removed from the oocytes by vigorous pipetting in the presence of 0.1% (w/v) hyaluronidase. Oocytes with an evenly granulated ooplasm and an extruded first polar body were selected and placed into the micromanipulation medium drop (containing donor cells and 7.5 µg/mL cytochalasin B) on a 60-mm cell culture dish (NUNC) covered with mineral oil.

Matured oocytes were enucleated by aspirating the first polar body and a portion of the adjacent cytoplasm (presumably containing the metaphase II plate) using a sharp-beveled glass pipette (WPI, Sarasota, Florida, USA) with a diameter of 20–25 µm. After enucleation, a donor cell was injected into the perivitelline space, with care taken to maximize the amount of cell membrane contact between the donor cell and the oocyte. The fusion and activation of nuclear transferred embryos were performed simultaneously using electrical pulses (two successive DC pulses of 1.2 kV/cm for 30 μs; BTX2000, BTX Inc., San Diego, CA, USA) in a fusion medium (0.3 M mannitol, 1.0 mM CaCl_2_, 0.1 mM MgCl_2_, 0.5 mM HEPES containing 0.3% [w/v] BSA). After fusion and activation, reconstructed embryos were placed into PZM-3 medium containing 0.3% (w/v) BSA and cultured at 38.5 °C in a humidified atmosphere of 5% (v/v) CO_2_ in air. Fusion was confirmed 40–60 min later, and fused embryos were maintained in culture until embryo transfer.

For embryo transfer, 200–300 cloned embryos were cultured *in vitro* from day 0 to day 1 and then surgically transferred into the oviductal ampullary-isthmic junction of surrogate sows exhibiting natural estrus (within one day of the onset of estrus). Pregnancy was diagnosed by ultrasonography, and the pregnant surrogates were delivered by natural parturition on day 114 to 120 of gestation (SCNT was performed on Day 0).

### *SCNA* mRNA-coding sequence analysis

One genotype-confirmed mutant minipig was used for *SCNA* mRNA-coding sequence analysis. Total RNA was isolated from brain tissue using a MiniBEST Universal RNA Extraction Kit (Takara). cDNA was synthesized from 1 μg of total RNA using a PrimeScript II 1st Strand cDNA Synthesis Kit (Takara) with 20 μL reaction volumes according to the manufacturer’s protocol. The resulting cDNA products were used as the template for amplification by PCR with primers specific to the sequence of porcine *SCNA* mRNA (PF: 5′-TGTGATCCAGGAACAGCTGT-3′ and PR: 5′-ACGTCTGTCAGCAGATCTCA-3′). The PCR products were collected for DNA sequencing to confirm whether the desired gene editing had occurred at the transcription level.

### Brain tissue collection, fixation and section

Collection and preparation of brain tissue were performed as previously described^26,36,50,51^. Briefly, minipigs were deeply anesthetized by intraperitoneal injection of sodium pentobarbital solution (100 mg/kg). A midventral sternal thoracotomy was performed, and a cannula was inserted in the aorta through the left ventricle. Then, the right atrium was opened, and normal saline (3 L) was injected through the cannula by gravity flow, followed by perfusion with 5 L ice-cold 4% paraformaldehyde in 0.1 M PBS solution. Next, the fixed brain was quickly extracted and cut into 3-mm-thick coronal blocks, immediately followed by overnight incubation at 4 °C in 4% paraformaldehyde-PBS solution. Fixed mesencephalon tissue from approximately the same anatomic parts of mutant and wild-type pigs were collected and cryoprotected by sequential overnight incubations in 10%, 20%, and 30% (w/v) sucrose-PBS solutions at 4 °C. After cryoprotection, mesencephalon tissue was cryosectioned into 40-µm-thick coronal sections using a SM 2000R sliding microtome (Leica, Nussolch, Germany). Free-floating sections were preserved in PBS containing 30% (w/v) sucrose and 30% (v/v) ethylene glycol at −20 °C.

### Immunohistochemical analysis

For immunohistochemical detection of α-synuclein-immunopositive pathology, free-floating brain sections were washed in PBS and incubated in endogenous peroxidase inhibitor solution (PBS containing of 0.2% H_2_O_2_) for 20 min. After a wash in PBS, the sections were blocked in a solution containing 4% normal horse serum, 1% (w/v) BSA, and 0.4% (v/v) TritonX-100 in PBS for 1 h at room temperature, followed by incubation with a mouse monoclonal α-synuclein antibody (Life #180215; diluted at 1:1,000) at 4 °C overnight. After extensive washing with PBS, sections were incubated with biotinylated horse anti-mouse IgG antibody (Vector #BA-2000; diluted at 1:1,000) at room temperature for 1.5 h. After more washes in PBS, the sections were visualized with an ABC-HRP Kit (Vector Laboratories #PK-6100) and DAB staining according to the manufacturer’s protocol. Sections were mounted on slides and allowed to dry naturally. They then underwent a de-fat step with chloroform and were stained with hematoxylin, dehydrated with ethanol, and finally sealed with neutral balsam. Slides were observed under a Eclipse Ti microscope (Nikon, Tokyo, Japan) to check for α-synuclein-immunopositive pathology, and images were captured with a NIS-Elements Advanced Research image system (Nikon). Finally, the images were processed and analyzed using Photoshop CS5 software (Adobe Systems Inc., San Jose, USA).

Since tyrosine hydroxylase (TH) is a specific marker for dopaminergic neurons^26,52^, TH immunohistochemistry was performed to quantify dopaminergic neurons in the SN. The general procedure used for TH immunohistochemical staining was the same as for α-synuclein. The primary antibody was anti-tyrosine hydroxylase (Millipore #MAB318; diluted at 1:1,000), and a nickel-enhanced DAB staining was employed. For comparative analysis of dopaminergic neurons in the SNs of the mutant versus wild-type minipig, at least three sections (randomly selected from approximately the same anatomic parts of the mesencephalon) were examined as previously described^26,52^. The number of dopaminergic neurons was expressed as the mean ± standard deviation (SD) and analyzed with an unpaired, two-tailed Student’s *t*-test. Statistical analysis was conducted with GraphPad Prism 5 software (La Jolla, CA, USA), and a *P-*value of less than 0.05 was considered statistically significant.

## Electronic supplementary material

Supplementary Information
