# Supplementary material for: CRISPR/Cas9-Mediated Generation of Guangxi Bama Minipigs Harboring Three Mutations in α-Synuclein Causing Parkinson’s Disease
Source: Sci Rep. 2018 Aug 20;8:12420. doi: 10.1038/s41598-018-30436-3 (PMC6102220; doi:10.1038/s41598-018-30436-3)
Supplement: Supplementary file 1 — Supplementary Information [file 41598_2018_30436_MOESM1_ESM.pdf]

Supplementary Information

CRISPR/Cas9-Mediated Generation of Guangxi Bama Minipigs Harboring Three Mutations in α-Synuclein Causing Parkinson's Disease

Xiang-Xing Zhu[1,*,+], Yi-Zhi Zhong[2,+], Yao-Wen Ge[3], Ke-Huan Lu[1] & Sheng-Sheng Lu[1,*]

[1]State Key Laboratory for Conservation and Utilization of Subtropical Agro-Bioresources; Guangxi High Education Key Laboratory for Animal Reproduction and Biotechnology; College of Animal Science and Technology, Guangxi University, Nanning 530004, China.

[2]Guangxi Nanning Yanleshang Biotechnology Co. LTD, Nanning 530004, China.

[3]Wuhan ViaGen Animal Breeding Resources Development Company, Wuhan 430073, China.

[*]Correspondence and requests for materials should be addressed to S.S.L. (E-mail: shengshenglu@sina.com) or X.X.Z. (E-mail: zhu_xiangxing@126.com).

[+]These authors contributed equally to this work.

Contents:

Supplementary Table S1

Supplementary Fig. S1

Supplementary Fig. S2

Supplementary Fig. S3

Supplementary Fig. S4

Full-length gel picture of Fig. 1B

Full-length gel picture of Fig. 2B Upper gel

Full-length gel picture of Fig. 2B Lower gel

Full-length gel picture of Fig. 2D

Full-length gel picture of Fig. S2 Upper gel

Full-length gel picture of Fig. S2 Middle gel

Full-length gel picture of Fig. S2 Lower gel

Full-length gel picture of Fig. S3A Left gel

Full-length gel picture of Fig. S3A Middle gel

Full-length gel picture of Fig. S3A Right gel

Supplementary Table S1 Off-target sites used for analysis of off-target mutations in gene-edited cell colonies

Off-target No.	Chr.	Strand	Position	Sequence*	Score	Gene	Primers for PCR and sequencing	Amplicon (bp)
sgRNA	Chr. 8	1	138648226	ACCAAGGAAGGAGTGGTTCATGG	100	NCBI Gene ID: 641350	-	-
Off-target 1#	Chr. 1	-1	84002721	AACAGGGAAGGAGTGGTTCAGGG	5.722891566	None	PF: GATGGATAGATCTTGTCTGG<br>PR: CGATTCCAGGACTCTTAGAG	504
Off-target 2#	Chr. 18	-1	20227588	GAGATGGAAGGAGTGGTTCATGG	1.30580855	None	PF: GGATACACGTACATGCAGTC<br>PR: TCACCAAGGATCTCACCATG	451
Off-target 3#	Chr. 6	1	27376728	ACCAAGGTAGGAGTCAGTCAGAG	0.025390982	NCBI Gene ID: 397478	PF: TACAGTGCAGCTCAGAGAGG<br>PR: ACCTAGCATCCAGCTCAAAC	531

*Red letters are showing the unmatched nucleotides in off-target sequences aligned to sgRNA. PAM sequences are labeled with underline.

```
                                          30                          46   50
Homa sapiens  001 MDVFMKGLSK AKEGVVAAAE KTKQGVAEAA GKTKEGVLYV GSKTKEGVVH
Bama minipig  001 MDVFMKGLSK AKEGVVAAAE KTKQGVAEAA GKTKEGVLYV GSKTKEGVVH

                  51 53
Homa sapiens  051 GVATVAEKTK EQVTNVGGAV VTGVTAVAQK TVEGAGSIAA ATGFVKKDQL
Bama minipig  051 GVTTVAEKTK EQVTNVGEAV VTGVTAVAQK TVEGAGSIAA ATGFGKKDQL

Homa sapiens  101 GKNEEGAPQE GILEDMPVDP DNEAYEMPSE EGYQDYEPEA
Bama minipig  101 GKNEEGAPQE GILEDMPVDP DNEAYEMPSE EGYQDYEPEA
```

Supplementary Fig. S1 Alignment of α-synuclein protein sequences of human (Homa sapiens; NCBI Gene No. 6622) and Bama minipig. Caged residues are showing SNCA mutations (A30P, E46K, H50Q, G51D and A53T) causing human Parkinson's disease (PD). Notice that the porcine α-synuclein normally contains a threonine (T) at position 53, indicating A53T which cause PD in human will not be functional in pigs. Residues not evolutionary conserved are marked in red.

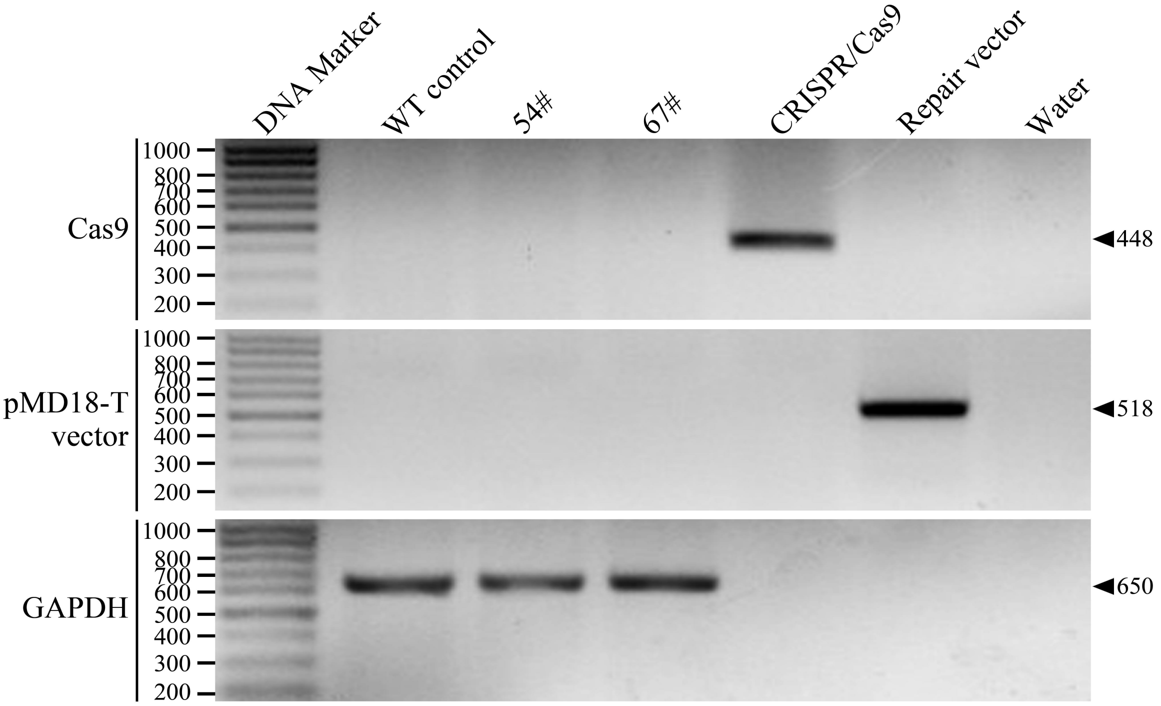

Supplementary Fig. S2 PCR amplification of Cas9 and repair vector confirmed no integration of exogenous genes in gene-edited cell colonies. Plasmids were represented as positive control, and wild-type (WT) cells were represented as negative control. GAPDH was used to confirm the DNA quality of all the samples.

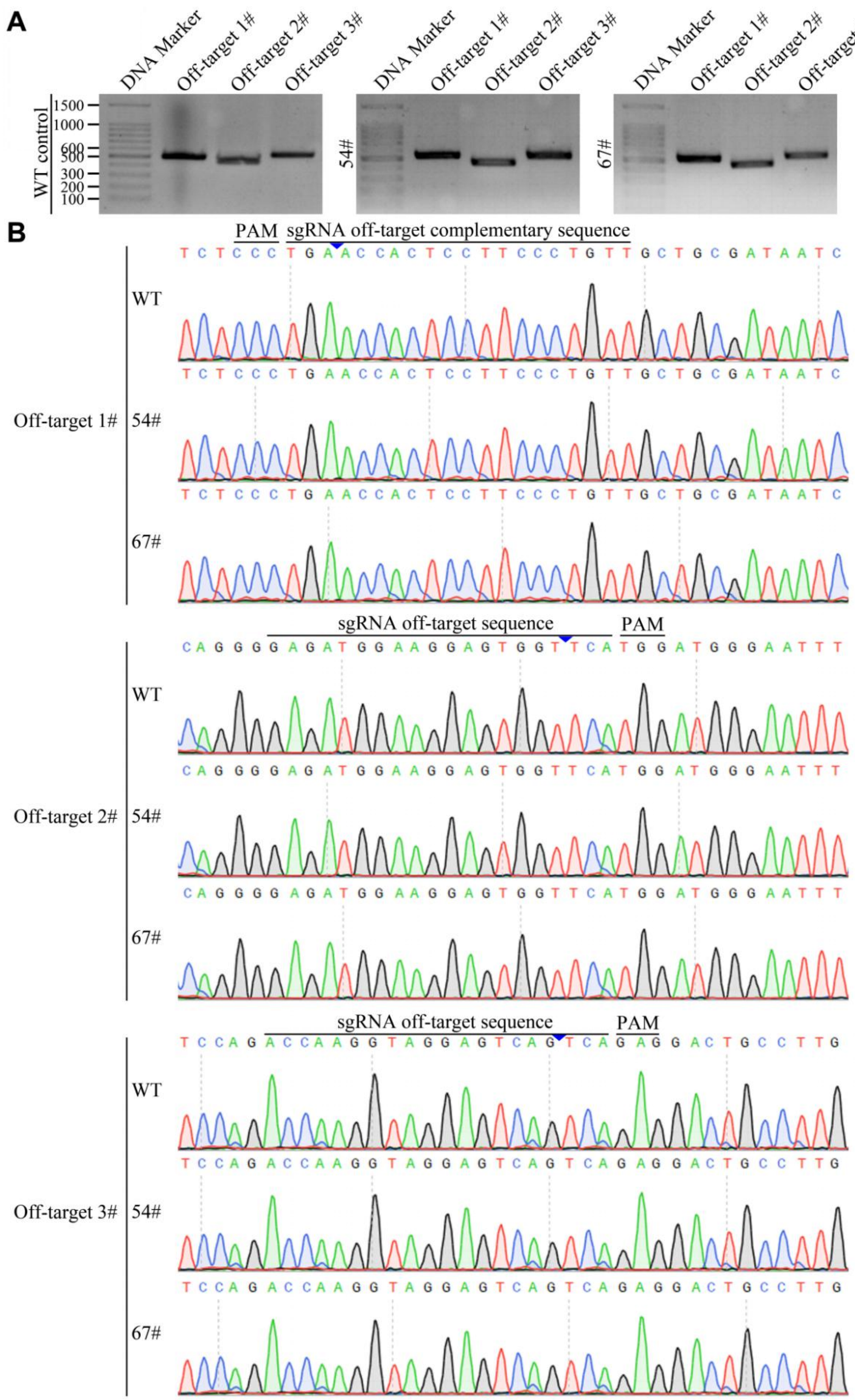

Supplementary Fig. S3 Analysis of the off-target effects by DNA sequencing. (A) PCR amplifications were

performed with two gene-edited cell colonies in three off-target loci (Off-target 1#~3#). (B) DNA sequencing suggested no mutations were occurred in all of the detected off-target loci in these two positive gene-edited cell colonies. The predicted cleavage sites in off-target sequences were marked with blue triangles.

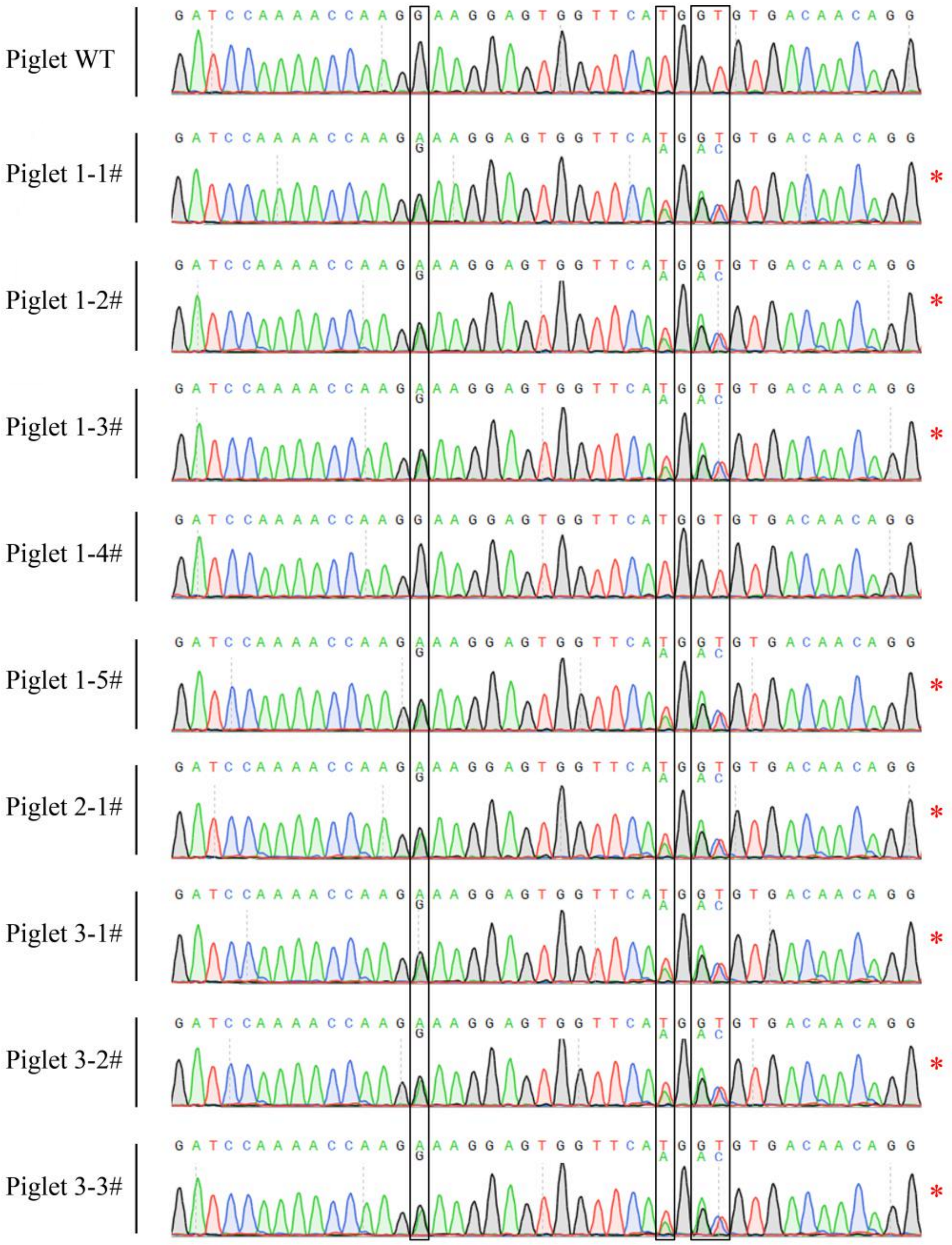

Supplementary Fig. S4 DNA sequencing confirmed that the cloned Guangxi Bama minipigs harboring designed mutations in *SCNA* locus. One wild-type (WT) pig was used as negative control, and 8 mutant minipigs were marked with red asterisks.

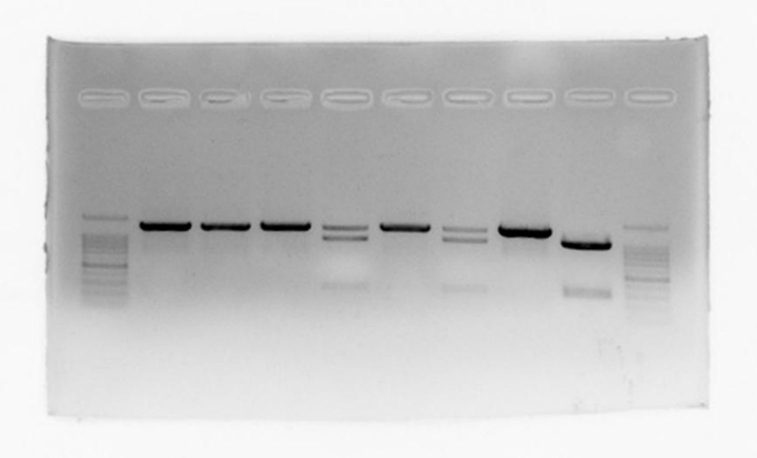

Full-length gel picture of Fig. 1B

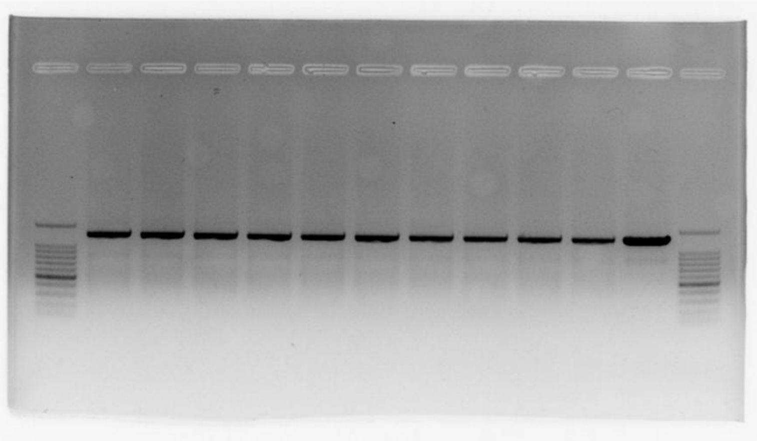

Full-length gel picture of Fig. 2B Upper gel

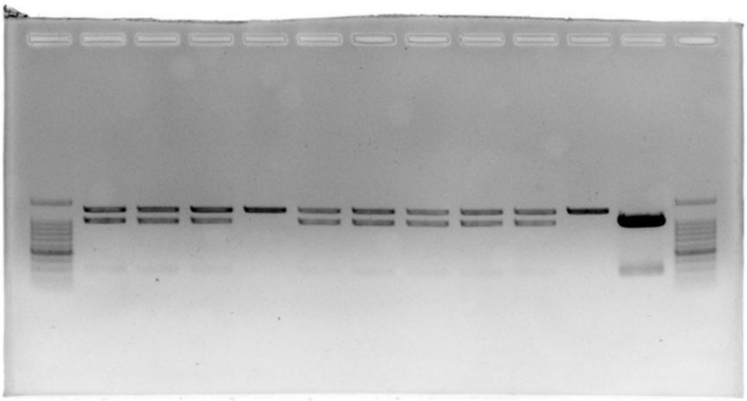

Full-length gel picture of Fig. 2B Lower gel

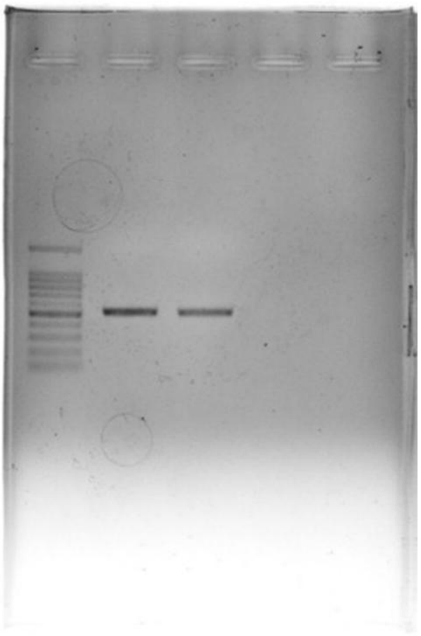

Full-length gel picture of Fig. 2D

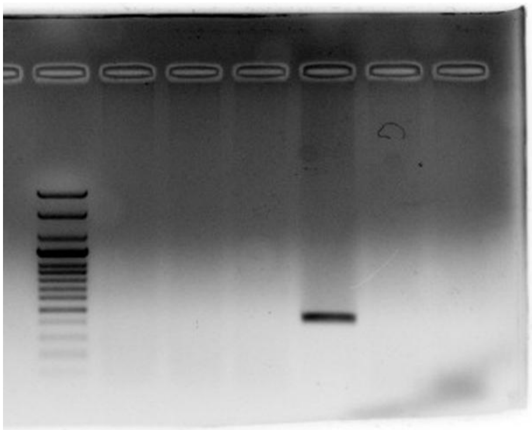

Full-length gel picture of Fig. S2 Upper gel

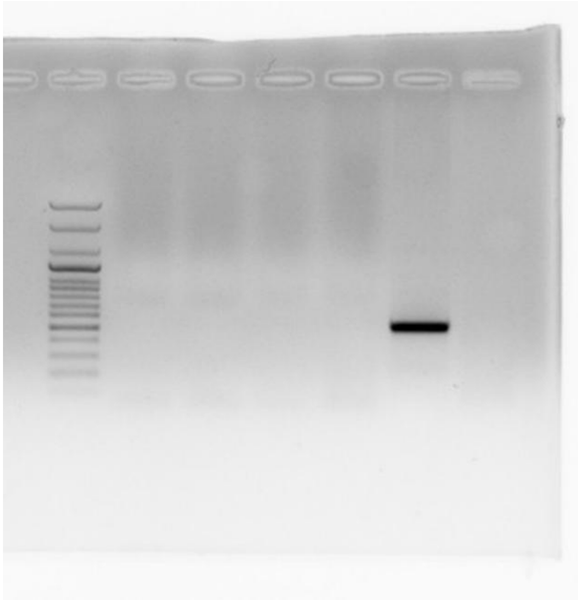

Full-length gel picture of Fig. S2 Middle gel

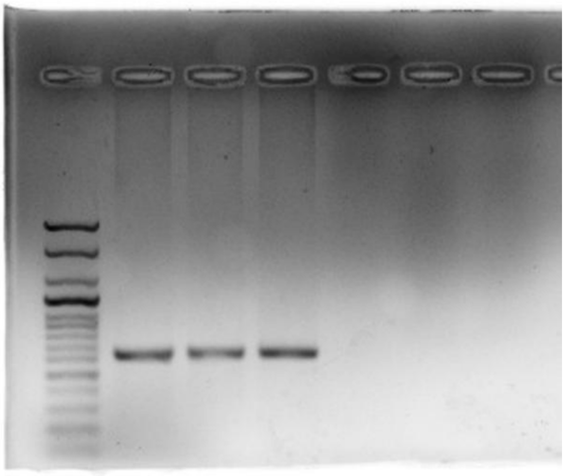

Full-length gel picture of Fig. S2 Lower gel

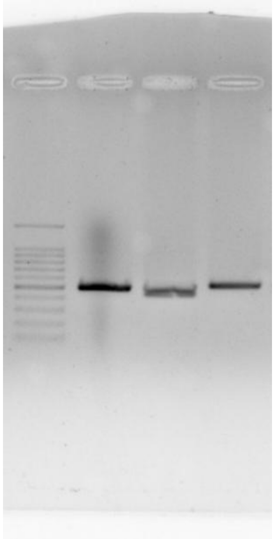

Full-length gel picture of Fig. S3A Left gel

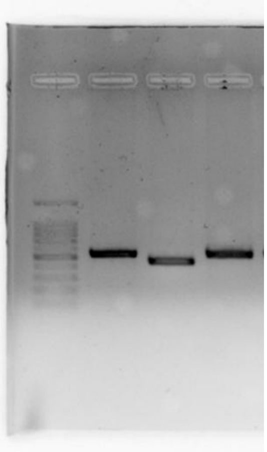

Full-length gel picture of Fig. S3A Middle gel

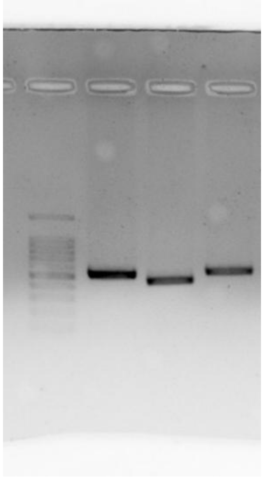

Full-length gel picture of Fig. S3A Right gel
